# Reliability and Validity of the Chinese Version of the Scale for Assessing the Stigma of Mental Illness in Nursing

**DOI:** 10.3389/fpsyt.2021.754774

**Published:** 2021-10-15

**Authors:** Weiwei Wang, Huixia Cui, Wenlu Zhang, Xiaoxiao Xu, Hong Dong

**Affiliations:** ^1^College of Nursing, Jinzhou Medical University, Jinzhou, China; ^2^Department of Radiation Oncology, The First Affiliated Hospital of Jinzhou Medical University, Jinzhou, China

**Keywords:** nursing, mental disorder, stigma, reliability, validity

## Abstract

**Background:** The prevalence of mental illness continues to increase in China, but research on stigma is still in its infancy, and there are even fewer studies on stigma among nurses. A comprehensive, effective and reliable tool is needed to assess stigma in nursing so that it can be reduced or eliminated to improve nursing quality. This study aimed to translate a 20-item scale for assessing the stigma of mental illness in nursing into Chinese and evaluate its reliability and validity.

**Methods:** An improved Brislin translation model was used to translate the nursing mental illness stigma assessment scale into Chinese. Content and face validity were determined by a panel of experts. A convenience sample of 501 nursing students was chosen. Confirmatory factor analysis, concurrent validity and known group comparison were used to evaluate the scale's structural validity. The reliability was evaluated based on the internal consistency reliability and 2-week retest reliability.

**Results:** The content validity index was 0.90. Confirmatory factor analysis showed that this study supported the three-factor model. The moderate correlation between the Chinese version of the Scale for Assessing the Stigma of Mental Illness in Nursing and the Perceived Devaluation Discrimination Scale suggested acceptable concurrent validity. Cronbach's α (0.863) and the retest coefficient (0.839) were indicative of internal consistency.

**Conclusion:** The Chinese version of the Scale for Assessing the Stigma of Mental Illness in Nursing has acceptable concurrent validity, marginal factor validity, and satisfactory reliability in China. Therefore, the three-factor structure of the Chinese scale should be considered.

**Relevance to Clinical Practice:** The Chinese version of the Scale for Assessing the Stigma of Mental Illness in Nursing can be used to understand the degree of mental illness stigma in nursing.

## Introduction

Stigma, a concept first proposed in 1963 by sociologist Goffman, is defined by him as follows: “the situation of the individual who is disqualified from full social acceptance” (1). Mental illness stigma, which is mainly produced in response to the abnormal behavior of people with mental illness, is an exclusionary attitude and leads to patients with mental disease patients feeling shame, inferiority and emotional distress (2, 3). Studies have shown that stigma negatively affects empowerment, social inclusion, quality of life, help seeking behaviors and adherence to treatment in people with mental illness (4, 5). Nursing staff, who have close contact with patients in the treatment of mental illness, can also discriminate against patients, stereotyping those with mental illness as incompetent, unpredictable, destructive, and violent, which undermines nursing quality (6, 7). This stigma can lead to a decline in the quality of nursing, and patients may experience isolation, abuse in healthcare, diagnostic overshadowing and exclusion (8, 9). It can also hinder the rehabilitation and social integration of patients with mental disorders and reduce the treatment effect in patients (10). Associated stigma may also affect nursing staff (11), leading them to perpetuate the cycle of exclusion in their clinical practice (12) or even to act in an extreme way (13). Stigma also affects nursing staffs' job satisfaction, interpersonal relationships, patient self-stigma, and nursing staff' professional identity (14). Correct assessment of nurses' stigma toward mental illness and effective adjustment to reduce and weaken this stigma can promote the recovery of patients and improve their clinical symptoms (15).

## Background

The World Health Organization reported in 2014 that one out of every four people may suffer from mental illness (16). Globally, an estimated 264 million people suffer from depression, 45 million suffer from bipolar disorder, and more than 20 million suffer from schizophrenia (17). In China, the number of people with mental illness is on the rise, with the prevalence rising from 3.2% in the 1970s to 15.56% (18). According to one study, China registered 4.3 million severely mentally ill patients in 2014 (19) while another study showed that in 2013, the number of such patients reached 16 million (20). Eight million were diagnosed with schizophrenia in 2018 (21). Mental health has become a prominent public health and social problem hindering China's economic development. At present, most of the research on mental illness stigma focuses on measuring the degree of stigma in the public and its related factors. Questionnaires used in these studies included the Struening Devaluation Scale (22) the Mass Discrimination Scal (23), the Link Perceived Devaluation Discrimination Scale (24) the Psychiatric Attitude Questionnaire (25, 26), and the Attitude Style Questionnaire (27). The objects of measurement of these questionnaires were the general public. Some questionnaires were used to measure the self-stigma of patients (28, 29) and the associated stigma of family members (30, 31). Only in a few articles were nursing undergraduates and doctors the objects of study (32, 33). There is no specific assessment scale for caregivers. At the same time, since the three principles of stigmatizing interventions (contact, education, and protest) are now integrated into mental health programs (34, 35), it is particularly important to have reliable tools that can objectively assess the effectiveness of these interventions (36). Based on the above situation, it is necessary for China to adopt the Mental illness Stigma assessment scale for Chinese nurses and verify its reliability and validity. Spanish scholars Dr. Sastre-Rus, Meritxell et al. developed a new tool for assessing the stigma of mental illness in nursing in 2020 (37). Therefore, the purpose of this study was to translate and localize the new scale, which was based on Peplau's psychodynamic nursing theory, and to measure its reliability and validity. The study was also to provide a way to quickly and accurately measure the degree of stigma of mental illness among nursing staff in China.

## Methods

### Study Design

This was a cross-sectional and observational survey designed to test the reliability and validity of the new Chinese version of the Scale for Assessing the Stigma of Mental Illness In Nursing tool, also known as the SASMIN.

### Setting

The participants in this research were senior college nursing students from the Nursing College of Jinzhou Medical University in China, and 501 students were recruited as volunteers.

### Instruments

#### SASMIN

The SASMIN is a 20-item scale developed by Dr. Sastre-Rus, Meritxell et al. to comprehensively assess the stigma of mental illness among nurses. SASMIN includes three dimensions: Factor 1: Violence/Dangerousness (8 items), Factor 2: Disability (5 items), and Factor 3: Irresponsibility/Lack of Competence (7 items). Both positive and negative wording is used (items 2, 3, 5, 10, 13, and 15 are forward scored, with the rest of the items are reverse scored). Each item is rated on a five-point Likert scale (5 = totally agree; 4 = somewhat agree; 3 = neutral; 2 = somewhat disagree; 1 = totally disagree). The total and dimension scores are the sum of the scores of each item, with the total score being between 20 and 100. A higher score infers a weaker degree of stigma. The original scale has acceptable internal consistency, with a Cronbach's alpha of 0.825 for the overall scale and 0.626–0.731 for the subscales.

#### Perceived Devaluation Discrimination

In this study, the Perceived Devaluation Discrimination (PDD) Scale was used to assess criterion validity (24, 38). The PDD mainly measures the perceived disparagement and discrimination mental patients and their family members receive from the general public to evaluate the perceived stigma of patients and their families. The scale also measures the level of public and social perceptions of stigmatization and discrimination against people with mental illness. The Chinese version of the scale, which was translated and localized by Xu Hui (39) in 2007, has been verified to have good reliability and validity. This scale is a self-rating scale consisting of 2 dimensions and 12 items. The author took the midpoint of 2.50 as the standard and compared the average score with it. Those with a score higher than 2.50 were considered to have stigma, while those with a score lower than 2.50 were considered to have no stigma.

#### Translation

We followed a modified Brislin translation model (40) consisting of (A) forward translation, (B) back translation and (C) revision. The English version of the scale was independently translated by two nursing graduate students who were proficient in English and had passed the CET-6. The Chinese version was independently translated into English by two nursing researchers with PhDs who were not familiar with the original SASMIN and were also proficient in English. Finally, two other bilingual experts were invited to perform a comparative analysis between the two translated versions and the original scale, and the items with large differences were re-translated and translated back. After the development of the third version, 10 students were recruited to conduct a pre-investigation on this version to ensure the quality of the scale before the reliability and validity tests. According to the subjects' understanding of the contents of the scale, information about the time required to complete the questionnaire, possible problems and suggestions, and ambiguous and difficult items was collected, and the researchers continued to make timely modifications and adjustments to produce the final Chinese version of the SASMIN.

### Data Collection

The researchers themselves distributed paper questionnaires to nursing students at Jinzhou Medical University individually from November to December 2020 and explained the content, purpose and significance of the questionnaires to the students before issuing them, reminding them to fill out the questionnaires carefully. After obtaining informed consent from the students, the questionnaires were distributed to the students on the spot, and the students completed the questionnaires independently. It took 5–10 min to complete the questionnaires, and the questionnaires were returned on the spot after completion. The researchers checked whether there were omissions and errors in the completed questionnaires and confirmed or revised the questionnaires with the students at once to ensure the completeness and quality of the questionnaire completion. Then, 31 randomly selected students were invited to complete the questionnaires again 2 weeks later to test the reliability of the retest. The retest interval is generally 10–14 days (41), so this interval was 14 days to ensure the reliability of the retest (41).

### Ethical Considerations

Ethical approval for this study was obtained from our institutional ethics committee. The SASMIN scale was authorized by the original author, and participants' informed consent was obtained. Data confidentiality was ensured during the survey. All the students signed a written informed consent form, and the study data were kept in a completely confidential way.

### Statistical Analysis

EpiData data entry software was used to double check the data entry, and SPSS 26.0 and AMOS 23.0 were used for statistical analysis (IBM).

An expert team consisting of 6 relevant experts (1 psychological expert, 1 doctor of nursing, 2 clinical nursing experts, and 2 nursing education experts) was invited to evaluate the content validity of the SASMIN scale. They had been working for 27 years on average. They rated each item with a four-point Likert scale (1 = not relevant, 2 = weakly relevant, 3 = strongly relevant, 4 = highly relevant). The content validity index (I-CVI) for each project was calculated based on the percentage of protocols rated 3 or 4 out of the total number. When the I-CVI is ≥0.78, it indicates that the overall content validity of the scale is good (42).

Descriptive analysis was used for the general information of the participants, and confirmatory factor analysis (CFA) was conducted to investigate the underlying factor structure of the translated scale. To confirm the replicability of the first-order three-factor structure of the SASMIN (43), the following indexes must be good: χ^2^/DF, goodness-of-fit index (GFI), adjusted goodness-of-fit index (AGFI), incremental fit index (IFI), Tucker-Lewis index (TLI), and comparative fit index (CFI). Generally, χ^2^/DF is required to be <3, while all of other values are required to be >0.9, indicating good adaptability of the model; however, a value >0.8 indicates that the model is acceptable. In addition, the root mean square error of approximation (RMSEA) should be <0.08, indicating good adaptability and good model fit.

By calculating the Cronbach's α coefficient, the retest reliability and the revised item-total correlation, the internal consistency reliability of the scale was tested. The minimum acceptable Cronbach's α coefficient was set to 0.7 (44). The adjusted item-total correlation represents the correlation between each item and the sum of the other items in the scale, which is evaluated using the standard of 0.3 (45).

Calibration correlation refers to using a recognized valid scale as a standard to test the degree of correlation between the measured scale and the standard scale. In this study, the PDD scale was used as the calibration standard for evaluation. Pearson correlation analysis was used to test the correlation between the Chinese version of the SASMIN scale and the PDD scale. When the correlation coefficient *r* > 0.7, the validity of the test is high. When 0.4 < *r* < 0.7, the validity of the test was moderate. When *r* < 0.4, the validity of the test is low (46).

For the discriminant validity, according to the total score of the SASMIN scale, the top 27% of scores were grouped as high and the bottom 27% as low, and the scores of both groups were analyzed using the two-tailed independent samples *t*-test. If the scores of the two groups reached the level of significance (*P* < 0.05), the discriminant validity was good.

To assess the degree of stigma, the total stigma score of each participant was calculated and then converted to a Z-score in the data (*N* = 501). The Z-score is also called the standard score, which is the difference between an original score and the mean divided by the standard deviation. Stigma was divided into four grades, namely, severe stigma, moderate stigma, mild stigma and no stigma, so each grade contained an interval of 1.5 standard deviations. A Z score below −1.5 is considered to indicate that there is very high stigma. A Z score between −1.5 and the mean is considered to indicate moderate stigma. A Z score that is above average but below 1.5 is considered to indicate mild stigma. Finally, a Z score higher than 1.5 is classified as no stigma. Therefore, the standardized scores were divided according to the principle that the higher the score is, the weaker stigma.

## Results

### Participant Characteristics

A total of 518 questionnaires were sent out, and 501 remained after the invalid questionnaires were removed, for a recovery rate of 96.72%. The respondents were college nursing students aged 18–25 years, and the average age was 20.94 ± 1.368 years. According to the results of the survey, the majority of respondents were female (442/501), which was mainly due to the lack of male students in the nursing student population. Eleven percent (55/501) of the students participated in mental illness-related extracurricular or community activities in college, 34.3% (172/501) of the students had contact with patients with mental illness, and 24.6% (123/501) of the students wanted to become psychiatric nurses. The detailed results are shown in Table 1.

**Table 1:** Participant characteristics.

**Characteristic**	**Group**	***n***	**%**
Gender	Male	59	11.8
	Female	442	88.2
Location	Rural	281	56.1
	City	220	43.9
Are you willing to become a psychiatric nurse	Willing	123	24.6
	Not willing	378	75.4
Have you participated in activities related to mental illness	Yes	55	11
	No	446	89
Whether a family member has a mental illness	Yes	22	4.4
	No	479	95.6
Have you ever met a mentally ill person	Yes	172	34.3
	No	329	65.7

### Content Validity

The contents validity index (I-CVI) of the items ranged from 0.830 to 1.000, and the contents validity index (S-CVI) of the scale was 0.975. Correlation analysis results showed that the scores of each item were positively correlated with the total scores, and the differences were statistically significant (*P* < 0.01).

### Construct Validity Analysis

**Figure 1:**
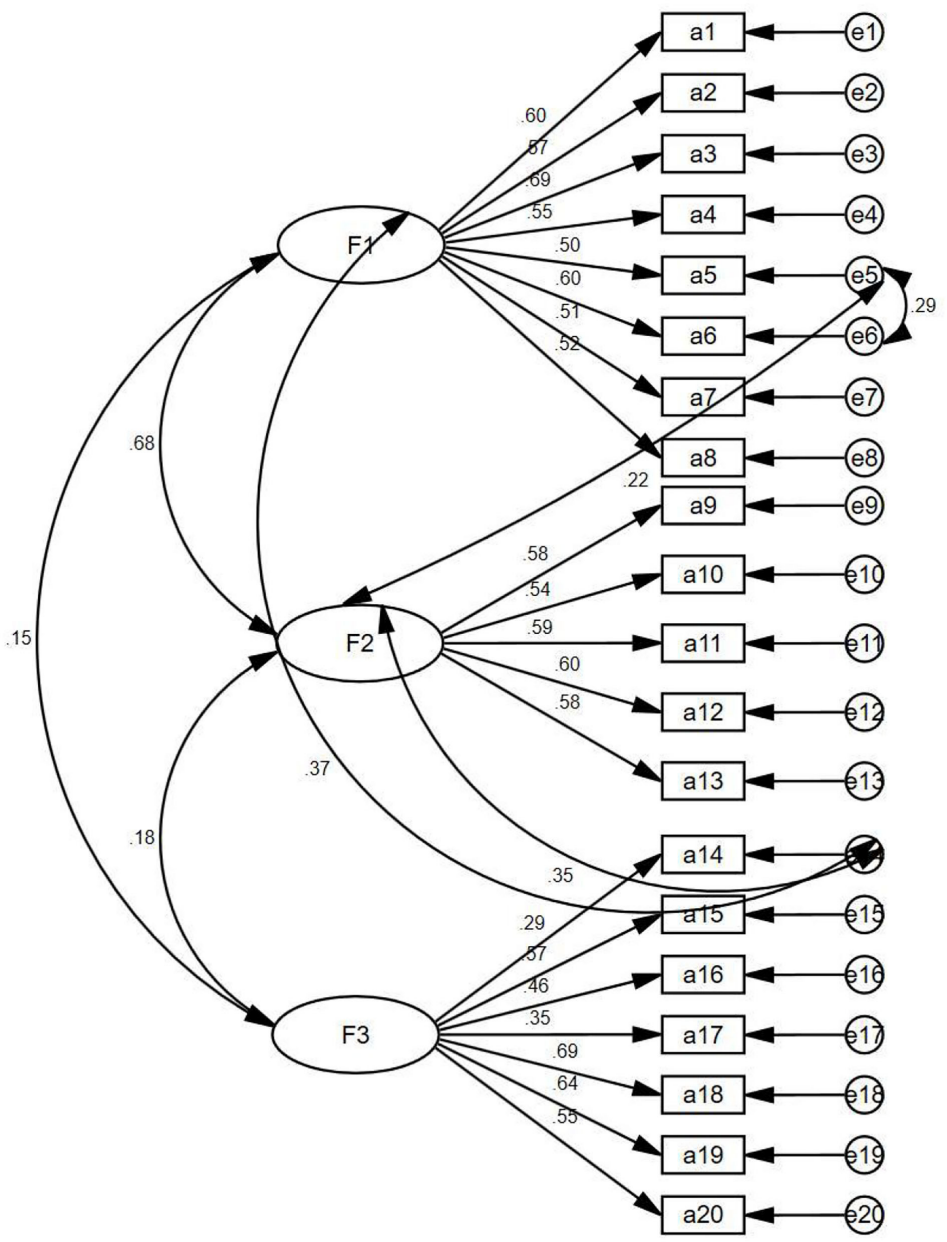
Standardized three-factor structural model of the SASMIN (*n* = 501).

In the model fitness index, the Chi-square degree of freedom was 2.388, the adjusted GFI was 0.917, the incremental fit index (IFI) was 0.899, the TFI was 0.881, the CFI was 0.898, and the root mean square error of approximation (RMSEA) was 0.053, which proved that the structural equation model of the Chinese version of the scale was valid and the Chinese data fit the original three-dimensional structural model well. The standardized factor load of each item was >0.5 (shown in Table 2), indicating that each item could explain its dimension, except items 1 (a14) and 5 (a17). Item 1 is that people with a mental disorder area burden on their family and society. Item 5 is that caring for a patient with a mental disorder is no more burden than caring for other patients. The standardized three-factor structural model of the SASMIN (*n* = 501) is shown in Figure 1.

**Table 2:** Factor loading of each item in the Chinese version of the scale.

**Item**	**F1**	**F2**	**F3**
6 (a1)	0.600		
7 (a2)	0.572		
9 (a3)	0.688		
11 (a4)	0.548		
12 (a5)	0.501		
14 (a6)	0.599		
16 (a7)	0.512		
17 (a8)	0.517		
4 (a9)		0.580	
8 (a10)		0.537	
18 (a11)		0.592	
19 (a12)		0.596	
20 (a13)		0.585	
1 (a14)			0.288
2 (a15)			0.570
3 (a16)			0.460
5 (a17)			0.352
10 (a18)			0.685
13 (a19)			0.644
15 (a20)			0.551

### Criterion Validity

In this study, the Chinese version of the PDD scale was used as the calibration scale to analyse its correlation with the total score and the dimensions of the SASMIN scale. The results showed that the total scores of the two scales was positively correlated (*r* = 0.401, *P* < 0.01). The significant correlation coefficients of the different dimensions and the PDD scale were 0.296, 0.306, and 0.355, respectively (*P* < 0.01).

### Reliability Analysis

The reliability analysis results showed that the Chinese version of the SASMIN had ideal internal consistency, with an overall Cronbach's alpha coefficient of 0.863 and Cronbach's alpha coefficients of 0.901, 0.820, and 0.875 for the three factors. In addition, the item-total correlation coefficients ranged from 0.327 to 0.600, which were all higher than 0.3. Therefore, the 20 items were all integrated to the questionnaire (Table 3).

**Table 3:** Cronbach's alpha coefficient if the item was deleted and item-total correlation.

**Item**	**Item-to-total correlation coefficient (*t*)**	**Cronbach's α if item deleted**
6 (a1)	0.428	0.807
7 (a2)	0.478	0.804
9 (a3)	0.523	0.802
11 (a4)	0.422	0.807
12 (a5)	0.483	0.804
14 (a6)	0.491	0.803
16 (a7)	0.403	0.808
17 (a8)	0.394	0.809
4 (a9)	0.473	0.804
8 (a10)	0.360	0.811
18 (a11)	0.426	0.807
19 (a12)	0.406	0.808
20 (a13)	0.473	0.805
1 (a14)	0.441	0.806
2 (a15)	0.275	0.815
3 (a16)	0.244	0.816
5 (a17)	0.208	0.818
10 (a18)	0.293	0.814
13 (a19)	0.282	0.814
15 (a20)	0.177	0.820

### Retest Reliability

The intragroup correlation coefficient (ICC) was used to evaluate the retest reliability of the scale. The sample size was 31, the questionnaire recovery rate was 100%, the ICC value of the total scale was 0.760, and the ICC values of each dimension were 0.766 (*P* < 0.000), 0.468 (*P* < 0.008), and 0.575 (*P* < 0.001).

### Discriminant Validity

Discriminant validity is based on the total score of the SASMIN scale, which was ranked from high to low; the top 27% of the scores were grouped into the high-score group, and the bottom 27% of the scores were grouped into the low-score group. In this study, the critical value scores were 52 and 63, respectively, and the scores of each item in the 2 groups were analyzed by using a two-tailed independent samples *t*-test. The results showed that the score difference of each item in the 2 groups reached the level of significance (*P* < 0.05). The detailed results are shown in Table 4.

**Table 4:** Discriminant validity analysis of the Chinese version of the scale.

**Item**	**Low-score group** **(*n* = 143), mean (SD)**	**High-score group** **(*n* = 152), mean (SD)**	***t-*test (*df*)**	***P-*value**
6	2.04 (0.655)	3.02 (0.893)	−10.579 (266.878)	0.000
7	2.95 (1.035)	4.31 (0.704)	−13.105 (291.247)	0.000
9	2.14 (0.679)	3.47 (0.887)	−14.108 (260.898)	0.000
11	2.28 (0.774)	3.39 (1.003)	−10.460 (277.491)	0.000
12	2.48 (0.830)	3.93 (0.868)	−14.447 (293.000)	0.000
14	2.471 (0.901)	3.77 (0.862)	−13.092 (293.000)	0.000
16	2.32 (1.004)	3.50 (1.002)	−9.988 (282.568)	0.000
17	2.11 (0.780)	3.19 (1.083)	−9.619 (267.627)	0.000
4	2.17 (0.842)	3.43 (0.965)	−11.763 (278.627)	0.000
8	2.54 (0.821)	3.50 (0.910)	−9.406 (275.055)	0.000
18	2.63 (0.793)	3.59 (0.853)	−9.830 (293.000)	0.000
19	2.56 (0.811)	3.61 (0.858)	−10.710 (293.000)	0.000
20	3.04 (0.870)	4.37 (0.689)	−14.358 (293.000)	0.000
1	2.95 (0.999)	4.27 (0.827)	−12.294 (293.000)	0.000
2	2.85 (0.988)	3.73 (0.996)	−7.509 (293.000)	0.000
3	2.38 (0.906)	3.11 (.916)	−6.852 (291.228)	0.000
5	2.36 (0.911)	2.94 (0.845)	−5.653 (293.000)	0.000
10	3.13 (0.844)	3.93 (0.836)	−8.035 (293.000)	0.000
13	3.08 (0.820)	3.80 (0.819)	−7.451 (293.000)	0.000
15	3.84 (0.873)	4.37 (0.878)	−5.093 (293.000)	0.000

### The Degree of Stigma

The Z score range of the Chinese version of the SASMIN was −3.44–3.05, with an average of 0. Specifically, 6.19% (31/501), 46.51% (233/501), 41.32% (207/501), and 5.99% (30/501) of participants were in the no stigma, low level of stigma, mild level of stigma and high stigma groups, respectively. Table 5 shows the average SASMIN score, which were normally distributed according to the skewness and kurtosis graphs. The total mean was 62.884, SD = 8.938, and the means of each dimension were 23.916, SD = 5.008; 15.764, SD = 3.164; and 23.204, SD = 3.873.

**Table 5:** Mean (SD) scores and skewness and kurtosis values of the scale.

**SASMIN items**	**Mean (SD)**	**Skewness**	**Kurtosis**
Violence/dangerousness	23.916 (5.008)	0.381	0.066
6.	2.57 (0.835)	0.494	0.553
7.	3.70 (1.019)	−0.490	−0.458
9.	2.85 (0.900)	0.390	0.022
11.	2.82 (0.984)	0.316	0.248
12.	3.28 (1.002)	−0.058	−0.526
14.	3.13 (1.003)	0.056	−0.428
16.	2.94. (1.054)	0.251	−0.533
17.	2.64 (1.054)	0.556	−0.328
Disability	15.765 (3.146)	0.674	0.129
4.	2.77 (0.990)	0.407	−0.285
8.	3.07 (0.902)	0.109	0.037
18.	3.15 (0.854)	0.034	0.620
19.	3.09 (0.907)	0.043	−0.281
20.	3.68 (0.948)	−0.380	−0.376
Irresponsibility and lack of competence	23.204 (3.873)	0.211	−0.125
1	3.59 (1.031)	−0.328	−0.542
2.	3.28 (1.003)	−0.260	0.018
3.	2.76 (0.923)	−0.024	−0.251
5.	2.65 (0.851)	0.246	0.151
10.	3.50 (0.885)	−0.401	0.162
13.	3.40 (0.848)	−0.307	0.239
15.	4.03 (0.905)	−1.014	1.343

## Discussion

This study was the first to translate the SASMIN into Chinese and test its reliability and validity. In the process of forming the Chinese version of the SASMIN, we strictly implemented the relevant process. Testing showed that the scale has good structural validity, discriminant validity and reliability and that it is reliable and effective in assessing the degree of nursing mental illness stigma in China. It can be used to measure nursing students' stigma of mental illness and could provide a reference for nursing educators for teaching plan formulation. It can also be used as a tool for nursing managers to assess nurses' stigma of mental illness, develop timely measures and interventions, improve nursing quality, and optimize nursing services to achieve the purpose of nursing, namely, “promoting patient health.”

The original Spanish scale was tested on students. The subjects in this study were also nursing students, which were from Jinzhou Medical University in northern China. These students were about to become nursing workers, making them a representative sample.

The content validity of the Chinese version assessed by six nursing experts resulted in an S-CVI ranged from 0.830 to 1.000; on average, the I-CVI was 0.975, so the scale items show good content validity.

For the CFA, the results showed that most items had factor loads higher than 0.5, except for items 1 and 5, which had loads close to 0.3. This may be because nursing undergraduates have not had in-depth contact with patients or have less contact with patients with mental diseases. Most nursing undergraduates' understanding of patients is influenced by the positive narration of teachers and the negative narration of society or family, which leads to the unstable attitude of nursing students toward mental diseases. The IFI was 0.899, the TFI was 0.881, and the CFI was 0.898. These results showed that the current three-factor model was within the acceptable range, although it did not achieve good fitting results. This was consistent with the results of the original author, so the dimensions of the scale were maintained, and further investigation will be conducted with a sample of clinical nursing staff to verify the results of this analysis.

We also chose the PDD scale for the assessment of the concurrent validity index. The Chinese version of the PDD has good validity and is widely used in China for measurement of stigma in the general public. Therefore, the use of the PDD for the concurrent validity index is justified. According to the Pearson correlation analysis, the total correlation between the two scales was 0.401, showing a moderate positive correlation.

According to the results on the degree of stigma, 47.21% of the students had moderate or higher stigma regarding mental illness, which indicated that the mental illness stigma of Chinese nursing students was serious. This may be due to the fact, shown in Table 1, that most nursing students (89%) did not participate in activities related to mental illness, and 65.7% had no contact with mentally ill people. They were therefore not familiar with patients. The results in Table 1 also show that only 24.6% of students were willing to be psychiatric nurses. Therefore, nursing educators and administrators should be reminded to add related academic training or lectures to actively guide nursing students' attitudes toward mental illness and help them establish a correct mindset.

### Limitations

However, several limitations should be taken into account when interpreting the results of this study. First, the research subjects were not nursing staff but senior nursing students who were easy to recruit. Therefore, the representativeness of the sample may be somewhat lacking. Second, due to the self-report nature of the survey, bias was inevitable. In addition, due to the lack of research on this topic, it was difficult to develop a proper discussion and compare the results with previous studies. However, this also highlights the novelty, interest, and readability of this study.

## Conclusion

Following a rigorous translation and validation process, the SASMIN demonstrated acceptable construct validity in terms of concurrent validity, discriminant validity, and factorial validity and acceptable internal consistency and test–retest reliability in China. The results of this study also showed that nursing mental illness stigma is prevalent in China, suggesting that measures need to be established to reduce mental illness stigma in nursing and improve the quality of care.

### Relevance to Clinical Practice

The SASMIN can identify the degree of stigma associated with mental illness among nursing staff in China and can be used as a tool by nursing managers to measure the degree of stigma and improve quality of care and patient satisfaction. Nursing educators should also take the measurement results of the scale as a reference, adjust the curriculum in a timely manner, improve the quality of education, and cultivate outstanding nursing talent.

## Data Availability Statement

The raw data supporting the conclusions of this article will be made available by the authors, without undue reservation.

## Ethics Statement

Written informed consent was obtained from the individual(s) for the publication of any potentially identifiable images or data included in this article.

## Author Contributions

WW: identify research content, research route design, survey, data analysis, and draft writing. HC: study route design, provide resources, and revise draft. WZ: review draft and provide statistical guidance. XX: collect data and organize data. HD: data collection and statistical analysis. All authors contributed to the article and approved the submitted version.

## Conflict of Interest

The authors declare that the research was conducted in the absence of any commercial or financial relationships that could be construed as a potential conflict of interest.

## Publisher's Note

All claims expressed in this article are solely those of the authors and do not necessarily represent those of their affiliated organizations, or those of the publisher, the editors and the reviewers. Any product that may be evaluated in this article, or claim that may be made by its manufacturer, is not guaranteed or endorsed by the publisher.

## References

[1] Goffman E. Stigma: notes on the management of spoiled identity. Postgrad Med J. (1969) 45:527. 10.1136/pgmj.45.527.642

[2] Xu H Li Z. Research progress on stigma in patients with mental illness. Chin J Nurs. (2007) 42:455–8.

[3] Corrigan PW Watson AC. Understanding the impact of stigma on people with mental illness. World Psychiatry. (2002) 1:16−20. 16946807 PMC1489832

[4] Clement S Schauman O Graham T Maggioni F Evans-Lacko S Bezborodovs N. What is the impact of mental health-related stigma on help-seeking? A systematic review of quantitative and qualitative studies. Psychol Med. (2015) 45:11–27. 10.1017/S0033291714000129 24569086

[5] Fung KM Tsang HW Chan F. Self-stigma, stages of change and psychosocial treatment adherence among Chinese people with schizophrenia: a path analysis. Soc Psychiatry Psychiatr Epidemiol. (2010) 45:561–8. 10.1007/s00127-009-0098-1 19649752

[6] Brunero S Lamont S. Challenges, risks and responses. In: Elders R Nizette K O'Brien A editors. Psychiatric and Mental Health Nursing. Amsterdam: Elsevier (2016). p. 552–76.

[7] Seeman N Tang S Brown AD Ing A. World survey of mental illness stigma. J Affect Disord. (2016) 190:115–21. 10.1016/j.jad.2015.10.011 26496017

[8] Vaghee S Kashani Lotfabadi M Salarhaji A Vaghei N Hashemi BM. Comparing the effects of contact-based education and acceptance and commitment-based training on empathy toward mental illnesses among nursing students. Iran J Psychiatry. (2018) 13:119–27. 10.5812/ijpbs.9672 29997657 PMC6037580

[9] World Health Organization. Mental Health Problems: The Undefined and Hidden Burden. World Health Organization (2012).

[10] Rodríguez-Almagro J Hernández-Martínez A Rodríguez-Almagro D Quiros-García JM Solano-Ruiz MDC. Level of stigma among Spanish nursing students toward mental illness and associated factors: a mixed-methods study. Int J Environ Res Public Health. (2019) 16:4870. 10.3390/ijerph16234870 31816966 PMC6926928

[11] Park K Seo M. Care burden of parents of adult children with mental illness: the role of associative stigma. Compr Psychiatry. (2016) 70:159–64. 10.1016/j.comppsych.2016.07.010 27624436

[12] Bates L Stickley T. Confronting Goffman: how can mental health nurses effectively challenge stigma? A critical review of the literature. J Psychiatr Ment Health Nurs. (2013) 20:569–75. 10.1111/j.1365-2850.2012.01957.x 22906050

[13] Riffel T Chen SP. Exploring the knowledge, attitudes, and behavioural responses of healthcare students towards mental illnesses-A qualitative study. Int J Environ Res Public Health. (2019) 17:25. 10.3390/ijerph17010025 31861420 PMC6981946

[14] Sercu C Ayala RA Bracke P. How does stigma influence mental health nursing identities? An ethno.graphic study of the meaning of stigma for nursing role identities in two Belgian psychiatric hospitals. Int J Nurs Stud. (2015) 52:307–16. 10.1016/j.ijnurstu.2014.07.017 25192962

[15] Çapar M Kavak F. Effect of internalized stigma on functional recovery in patients with schizophrenia. Perspect Psychiatr Care. (2019) 55:103–11. 10.1111/ppc.12309 30019336

[16] WHO. Mental Health Atlas. (2014). Available online at: https://www.who.int/publications-detail-redirect/mental-health-atlas-2014

[17] GBD 2017 Disease and Injury Incidence and Prevalence Collaborators. Global, regional, and national incidence, prevalence, and years lived with disability for 354 diseases and injuries for 195 countries and territories, 1990-2017: a systematic analysis for the Global Burden of Disease Study 2017. Lancet. (2018) 392:1789–858. 10.1016/S0140-6736(18)32279-7 30496104 PMC6227754

[18] China News. There are More Than 16 Million Serious Mental Patients in China, and Most Families are Destirpated. (2010). Available online at: https://www.chinanews.com/jk/jk-hyxw/news/2010/05-31/2314268.shtml (accessed August 5, 2021).

[19] China Health and Family Planning Commission of the PRC. National Mental Health Work Plan (2015-2020). (2015). Available online at: http://www.gov.cn/zhengce/2015-06/18/content_2881440.htm (accessed August 5, 2021).

[20] Metropolitan Express. There are about 16 million people with severe mental illness in China, causing more than 10,000 cases each year. Psychol Consult. (2013) 4:61.

[21] Wong YI Kong D Tu L Frasso R. “My bitterness is deeper than the ocean”: understanding internalized stigma from the perspectives of persons with schizophrenia and their family caregivers. Int J Ment Health Syst. (2018) 12:14. 10.1186/s13033-018-0192-4 29636792 PMC5883360

[22] Struening EL Perlick DA Link BG Hellman F Herman D Sirey JA. Stigma as a barrier to recovery: the extent to which caregivers believe most people devalue consumers and their families. Psychiatr Serv. (2001) 52:1633–8. 10.1176/appi.ps.52.12.1633 11726755

[23] Mak WW Cheung FM Wong SY Tang WK Lau JT Woo J. Stigma towards people with psychiatric disorders. Hong Kong Med J. (2015) 21(Suppl. 2):9–12. 25852095

[24] Bruce GL Struening EL Neese-todd S Asmussen S Phelan JC. On describing and seeking to change the experience of stigma. Am J Psychiatr Rehabil. (2002) 201–31. 10.1080/10973430208408433

[25] Gao S-Y Fei L-P. Attitudes towards mental illness among different groups. Chin Mental Health J. (2001) 107–9.

[26] Corrigan P Markowitz FE Watson A Rowan D Kubiak MA. An attribution model of public discrimination towards persons with mental illness. J Health Soc Behav. (2003) 44:162–79. 10.2307/1519806 12866388

[27] Ihalainen-Tamlander N Vähäniemi A Löyttyniemi E Suominen T Välimäki M. Stigmatizing attitudes in nurses towards people with mental illness: a cross-sectional study in primary settings in Finland. J Psychiatr Ment Health Nurs. (2016) 23:427–37. 10.1111/jpm.12319 27500395

[28] Gu XP Li L. Research progress on influencing factors and intervention of stigma in patients with schizophrenia. Nurs Res. (2019) 4285–9.

[29] Sanjuan AM. The stigma of mental disorders discrimination and social exclusion. Quad Psicol. (2011) 13:7–17. 10.5565/rev/qpsicologia.81633730906

[30] Gu J Li Z. Research progress on internal stigma associated with family members of patients with mental illness. Nurs Res. (2019) 33:3188–91.

[31] Mitter N Ali A Scior K. Stigma experienced by family members of people with intellectual and developmental disabilities: multidimensional construct. BJPsych Open. (2018) 4:332–8. 10.1192/bjo.2018.39 30140444 PMC6094883

[32] Jin XX. Investigation on stigma among 192 nursing staff on severe mental illness. China Health Industry. (2018) 15:185–6. 10.16659/j.cnki.1672-5654.2018.28.185

[33] Taguibao C Rosenheck R. Medical education and the stigmatization of mental illness in the Philippines. Cult Med Psychiatry. (2021) 45:312–31. 10.1007/s11013-020-09688-0 32930905

[34] Watson AC Corrigan PW. Challenging public stigma: a targeted approach. In: Corrigan PW editors. On the Stigma of Mental Illness: Practical Strategies for Research and Social Change. American Psychological Association (2005). p. 281–95.

[35] Hankir A Zaman R Evans-Lacko S. The Wounded Healer: an effective anti-stigma intervention targeted at the medical profession? Psychiatr Danub. (2014) 26(Suppl. 1):89–96. 10.1016/S0924-9338(15)31082-8 25413520

[36] Corrigan PW Powell KJ Michaels PJ. Brief battery for measurement of stigmatizing versus affirming attitudes about mental illness. Psychiatry Res. (2014) 215:466–70. 10.1016/j.psychres.2013.12.006 24388505

[37] Sastre-Rus M Tomás-Sábado J Juliá-Sanchis R Roldán-Merino JF Puig-Llobet M Lluch-Canut MT. Development and psychometric testing of a scale for assessing the associative stigma of mental illness in nursing. J Clin Nurs. (2020) 29:4300–12. 10.1111/jocn.15467 32808371

[38] Yu Q Zhai J Fan M Chen M Gao Y. A survey on stigma of depression among psychiatric and non-psychiatric health care workers. Chin J Neuropsychiatr Disord. (2014) 40:301−4.

[39] Xu H Li Z. A survey of the perception of psychiatric patients as belittled/discriminated by nursing undergraduates at a university. J Nurs. (2008) 8–10. 10.16460/j.issn1008-9969.2008.04

[40] Richard W. Brislin. Back-translation for cross-cultural research. J Cross Cult Psychol. (1970) 1:185–216. 10.1177/135910457000100301

[41] Minglong WU. Questionnaire Statistical Analysis Practice -SPSS Operation and Application. Chongqing: Chongqing University Press (2017). p. 158–93.

[42] Jingcheng S Xiankun M Zhenqiu S. Application of content validity index in scale compilation. J Central South Univ (Med Sci Ed). (2012) 37:152–5. 10.3969/j.issn.1672-7347.2012.02.007 22561427

[43] Annear MJ Otani J Li J. Japanese-language dementia knowledge assessment scale: Psychometric performance, and health student and professional understanding. Geriatr Gerontol Int. (2017) 17:1746–51. 10.1111/ggi.12911 27680686

[44] Chang Q Sha F Chan CH Yip PSF. Validation of an abbreviated version of the Lubben Social Network Scale (“LSNS-6”) and its associations with suicidality among older adults in China. PLoS ONE. (2018) 13:e0201612. 10.1371/journal.pone.0201612 30071067 PMC6072030

[45] Ferketich S. Focus on psychometrics. Aspects of item analysis. Res Nurs Health. (1991) 14:165–8. 10.1002/nur.4770140211 2047538

[46] Wang Y Sun AF. Statistical estimation of correlation validity of test criteria. Chin Med Guide. (2011) 9:234–5. 10.15912/j.cnki.gocm.2011.31.025

