# Supplementary material for: Reliability and Validity of the Chinese Version of the Scale for Assessing the Stigma of Mental Illness in Nursing
Source: Front Psychiatry. 2021 Oct 15;12:754774. doi: 10.3389/fpsyt.2021.754774 (PMC8555579; doi:10.3389/fpsyt.2021.754774)
Supplement: Supplementary file 1 [file Presentation_1.zip › 09.22frontiers in psychologyμèòτ¿┐μö»μÆæμ¥ÉμûÖ/chinese version scale.docx]

**护理人员精神疾病污名评估量表**

| **维度** | **量表条目** | **非常不同意** | **不同意** | **不确定** | **同意** | **非常同意** |
| --- | --- | --- | --- | --- | --- | --- |
| **暴力与危险** | **6.患有精神障碍的人比其他人有更大的暴力倾向。** |  |  |  |  |  |
|  | **7.患有精神障碍的人应该与社会隔离。** |  |  |  |  |  |
|  | **9. 有精神障碍的人更容易犯罪。** |  |  |  |  |  |
|  | **11. 所有进入精神病院的患者都应该先隔离一段时间。** |  |  |  |  |  |
|  | **12.所有精神病人都有失常的行为。** |  |  |  |  |  |
|  | **14.大多有精神障碍的人都很危险。** |  |  |  |  |  |
|  | **16.当我护理有精神障碍的病人时我感到害怕。** |  |  |  |  |  |
|  | **17.应该把精神障碍患者与其他病人隔离开。** |  |  |  |  |  |
| **残疾** | **4.患有精神障碍的人做事不考虑后果** |  |  |  |  |  |
|  | **8.一般来说，精神病人拒绝治疗性帮助。** |  |  |  |  |  |
|  | **18.所有精神障碍患者最终都会再次入院治疗。** |  |  |  |  |  |
|  | **19.精神障碍患者自己无法寻求帮助。** |  |  |  |  |  |
|  | **20. 所有进入精神病院的患者都需要进行身体约束。** |  |  |  |  |  |
| **不负责任和缺乏能力** | **1.精神障碍患者是家庭和社会的负担。** |  |  |  |  |  |
|  | **2.患有精神障碍的人可以和其他人一样成为一名优秀的专家。** |  |  |  |  |  |
|  | **3.有精神障碍的人可以担负起照顾孩子的责任。** |  |  |  |  |  |
|  | **5. 护理精神障碍患者和护理其他患者一样，没会有更多的负担。** |  |  |  |  |  |
|  | **10. 有精神障碍的人可以过正常的生活。** |  |  |  |  |  |
|  | **13. 与精神障碍患者共事是有收获的。** |  |  |  |  |  |
|  | **15. 有精神障碍的人和其他人一样享有同样的权利。** |  |  |  |  |  |
