# Supplementary material for: Reliability and Validity of the Chinese Version of the Scale for Assessing the Stigma of Mental Illness in Nursing
Source: Front Psychiatry. 2021 Oct 15;12:754774. doi: 10.3389/fpsyt.2021.754774 (PMC8555579; doi:10.3389/fpsyt.2021.754774)
Supplement: Supplementary file 1 [file Presentation_1.zip › 09.22frontiers in psychologyμèòτ¿┐μö»μÆæμ¥ÉμûÖ/Scale for Assessing the Stigma of Mental Illness I.docx]

Scale for Assessing the Stigma of Mental Illness In Nursing

|  | **Total**  **desacuerdo** | **Considerable desacuerdo** | **Neutro**  **(no acuerdo ni desacuerdo)** | **Considerable acuerdo** | **Total de acuerdo** |
| --- | --- | --- | --- | --- | --- |
| 1. Las personas con trastorno mental son una carga para su familia y para la sociedad. | 1 | 2 | 3 | 4 | 5 |
| 2. Las personas con trastornos mentales pueden ser tan buenos profesionales como el resto. | 1 | 2 | 3 | 4 | 5 |
| 3. Una persona con un trastorno mental puede responsabilizarse del cuidado de sus hijos o menores. | 1 | 2 | 3 | 4 | 5 |
| 4. Las personas con un trastorno mental actúan sin pensar en las consecuencias. | 1 | 2 | 3 | 4 | 5 |
| 5. El cuidado de un paciente con trastorno mental no me supone más carga que el cuidado del resto. | 1 | 2 | 3 | 4 | 5 |
| 6. Las personas con un trastorno mental tienen un potencial de conductas violentas superior al de otras personas. | 1 | 2 | 3 | 4 | 5 |
| 7. La mejor opción para la sociedad es aislar a los pacientes con trastornos mentales. | 1 | 2 | 3 | 4 | 5 |
| 8. En general las personas con trastorno mental rechazan ayuda terapéutica. | 1 | 2 | 3 | 4 | 5 |
| 9. Las personas con trastorno mental tienen más posibilidad de cometer actos delictivos. | 1 | 2 | 3 | 4 | 5 |
| 10. Las personas con trastorno mental pueden llevar una vida normal. | 1 | 2 | 3 | 4 | 5 |
| 11. Todos los pacientes que ingresan en una unidad de salud mental deben ser aislados las primeras horas. | 1 | 2 | 3 | 4 | 5 |
| 12. Todas las personas con trastorno mental tienen conductas inadecuadas. | 1 | 2 | 3 | 4 | 5 |
| 13. Trabajar con pacientes con trastorno mental es muy gratificante. | 1 | 2 | 3 | 4 | 5 |
| 14. La mayoría de las personas con trastorno mental son peligrosas. | 1 | 2 | 3 | 4 | 5 |
| 15. Los pacientes con trastorno mental tienen los mismos derechos que el resto. | 1 | 2 | 3 | 4 | 5 |
| 16. Me provoca miedo el cuidar a personas con un trastorno mental. | 1 | 2 | 3 | 4 | 5 |
| 17. Los pacientes con trastorno mental deben estar aislados del resto de pacientes. | 1 | 2 | 3 | 4 | 5 |
| 18. Todos los pacientes con trastorno mental acaban reingresando. | 1 | 2 | 3 | 4 | 5 |
| 19. Las personas con trastorno mental no son capaces de buscar ayuda por si solas. | 1 | 2 | 3 | 4 | 5 |
| 20. Todos los pacientes ingresados en unidades de salud mental precisan contención mecánica. | 1 | 2 | 3 | 4 | 5 |

| 1 | F3 | Ítem - |
| --- | --- | --- |
| 2 | F3 | Ítem + |
| 3 | F3 | Ítem + |
| 4 | F2 | Ítem - |
| 5 | F3 | Ítem + |
| 6 | F1 | Ítem - |
| 7 | F1 | Ítem - |
| 8 | F2 | Ítem - |
| 9 | F1 | Ítem - |
| 10 | F3 | Ítem + |
| 11 | F1 | Ítem - |
| 12 | F1 | Ítem - |
| 13 | F3 | Ítem + |
| 14 | F1 | Ítem - |
| 15 | F3 | Ítem + |
| 16 | F1 | Ítem - |
| 17 | F1 | Ítem - |
| 18 | F2 | Ítem - |
| 19 | F2 | Ítem - |
| 20 | F2 | Ítem - |

| F1 | PERSONA-VIOLENCIA-PELIGROSIDAD |
| --- | --- |
| F2 | SALUD-INCAPACIDAD |
| F3 | ROL-ENTORNO-RESPONSABILIDAD-COMPETENCIA |
